# The Interplay of Sirtuin-1, LDL-Cholesterol, and HDL Function: A Randomized Controlled Trial Comparing the Effects of Energy Restriction and Atorvastatin on Women with Premature Coronary Artery Disease

**DOI:** 10.3390/antiox11122363

**Published:** 2022-11-29

**Authors:** Dalila Pinheiro Leal, Gustavo Henrique Ferreira Gonçalinho, Thauany Martins Tavoni, Karen Lika Kuwabara, Ana Paula Paccanaro, Fatima Rodrigues Freitas, Célia Maria Cassaro Strunz, Luiz Antonio Machado César, Raul Cavalcante Maranhão, Antonio de Padua Mansur

**Affiliations:** 1 Faculdade de Medicina da Universidade de São Paulo, University of São Paulo, Sao Paulo 05508-060, Brazil; 2 Servico de Prevencao, Cardiopatia da Mulher e Reabilitacao Cardiovascular, Instituto do Coracao do Hospital das Clinicas da Faculdade de Medicina da Universidade de Sao Paulo (InCor-HCFMUSP), Sao Paulo 05403-900, Brazil; 3 Laboratorio de Metabolismo de Lipides, Instituto do Coracao do Hospital das Clinicas da Faculdade de Medicina da Universidade de Sao Paulo (InCor-HCFMUSP), Sao Paulo 05403-900, Brazil; 4 Laboratorio de Analises Clinicas, Instituto do Coracao do Hospital das Clinicas da Faculdade de Medicina da Universidade de Sao Paulo (InCor-HCFMUSP), Sao Paulo 05403-900, Brazil; 5 Unidade Clinica de Coronariopatias Cronicas, Instituto do Coracao do Hospital das Clinicas da Faculdade de Medicina da Universidade de Sao Paulo (InCor-HCFMUSP), Sao Paulo 05403-900, Brazil; 6 Faculdade de Ciencias Farmaceuticas da Universidade de Sao Paulo, University of São Paulo, Sao Paulo 05508-060, Brazil

**Keywords:** atherosclerosis, coronary artery disease, HDL, oxidation, atorvastatin, calorie restriction

## Abstract

Introduction: HDL function has gained prominence in the literature as there is a greater predictive capacity for risk in early coronary artery disease when compared to the traditional parameters. However, it is unclear how dietary energy restriction and atorvastatin influence HDL function. Methods: A randomized controlled trial with 39 women with early CAD divided into three groups (n = 13): energy restriction (30% of VET), atorvastatin (80 mg), and control. Analyses of traditional biochemical markers (lipid and glucose profile), circulating Sirt-1, and HDL function (lipid composition, lipid transfer, and antioxidant capacity). Results: Participants’ mean age was 50.5 ± 3.8 years. Energy restriction increased Sirt-1 by 63.6 pg/mL (95%CI: 1.5–125.7; *p* = 0.045) and reduced BMI by 0.8 kg/m^2^ (95%CI: −1.349–−0.273; *p* = 0.004) in a manner independent of other cardiometabolic factors. Atorvastatin reduced LDL-c by 40.0 mg/dL (95%CI: −69.910–−10.1; *p* = 0.010). Increased Sirt-1 and reduced BMI were independently associated with reduced phospholipid composition of HDL (respectively, β = −0.071; CI95%:−0.136–−0.006; *p* = 0.033; β = 7.486; CI95%:0.350–14.622; *p* = 0.040). Reduction in BMI was associated with lower HDL-free cholesterol (β = 0.818; CI95%:0.044–1.593; *p* = 0.039). LDL-c reduction by statins was associated with reduced maximal lipid peroxide production rate of HDL (β = 0.002; CI95%:0.000–0.003; *p* = 0.022) and total conjugated diene generation (β = 0.001; CI95%:0.000–0.001; *p* = 0.029). Conclusion: This study showed that energy restriction and atorvastatin administration were associated with changes in lipid profile, serum Sirt-1 concentrations, and HDL function.

## 1. Introduction

Coronary artery disease (CAD) is a leading cause of death in women [1]. Estrogens play an essential role in vascular health, and the decline of their circulating levels in menopause was associated with an increased risk of cardiovascular events in women [2,3]. However, CAD is less prevalent in premenopausal women, and the possible hypotheses are probably not attributed to direct plasma concentration estrogen-dependent mechanisms [1]. Therefore, premature CAD may involve other pathophysiological pathways.

Low plasma concentrations of high-density lipoprotein (HDL) cholesterol are an important risk factor for atherosclerosis and are prevalent in women with premature CAD [3]. Nevertheless, the predictive capacity of HDL-c is still not completely understood. Patients with similar HDL-c plasma concentrations may express different HDL function, usually measured by reverse cholesterol transport capacity [4]. Emerging evidence shows that HDL dysfunction (i.e., lower reverse cholesterol transport capacity) might predict cardiometabolic risk with higher precision [5]. Paraoxonase 1 (PON1), a component of HDL particles, reduces lipid peroxidation of other lipoproteins, and the antioxidant capacity of HDL depends mainly on its content [5]. Measuring lipid peroxidation products, namely conjugated dienes, is another approach to assessing HDL antioxidant capacity and therefore the lipoprotein’s function [6]. Furthermore, studies have shown that HDL lipid composition (phospholipids, triglycerides, esterified, and free cholesterol) and transfer are predictors of premature CAD [4,7].

Overall antioxidant defense influences HDL function [6]. One pivotal protein in antioxidant defense is sirtuin-1 (Sirt-1), which has been associated with better cardiometabolic risk factors control and longevity [8]. Its activity is increased mainly by energy restriction [8]. The effects of atorvastatin on antioxidant defense are known [9]; however, it is not known whether its mechanisms depend on Sirt-1. Furthermore, it is unclear if the increase in Sirt-1 modulates HDL antioxidant capacity as well as lipid composition and transfer.

Therefore, the objective of our study was to explore the effects of atorvastatin and energy restriction on circulating Sirt-1 and assess its relationship with HDL function measured by antioxidant capacity, particle lipid composition, and transfer.

## 2. Materials and Methods

### 2.1. Study Design, Participants, and Interventions

This randomized controlled trial allocated 39 pre-menopausal women with premature coronary artery disease (CAD) into three groups: energy restriction group, statin group, and control group. The study investigated the effects of 60-day energy restriction or atorvastatin on serum sirtuin-1 (Sirt-1), HDL particle lipid transfer, and antioxidant capacity.

Participants were recruited at the Instituto do Coracao do Hospital das Clinicas da Faculdade de Medicina da Universidade de Sao Paulo (InCor-HCFMUSP), Sao Paulo, Brazil. After all explanations, the participants signed the informed consent form. The Ethics Committee approved the study of Incor-HCFMUSP under the registration protocol 4413/016/079, which was in line with the Declaration of Helsinki, and the clinical trial was registered under the number NCT59937516.1.0000.0068.

As inclusion criteria, the following characteristics were considered: women with stable CAD (coronary lesion >70%) documented by coronary angiography, age ≤ 55 years, and body mass index (BMI) ≥25 kg/m^2^. As for the exclusion criteria, the following characteristics were considered: chronic kidney disease, liver failure, hypothyroidism, rheumatic diseases, type 1 diabetes mellitus, alcoholism, and recent surgery (≤6 months).

After recruitment, participants underwent a 45-day washout of statin treatment before starting the study. For the energy restriction group, statin treatment was suspended until the end of the study. After the washout period, interventions were started. For the participants of the statin treatment group, 80 mg of atorvastatin was prescribed.

As for the energy restriction group, a 30% energy restriction of the reported energy intake was prescribed. For food intake analysis, food diaries of 3 days (2 nonconsecutive weekdays and one day of the weekend for a more precise measure of eating habits). Food nutrient composition data were inputted into the AVANUTRI Software^®^ (Avanutri e Nutrição, Três Rios, Brazil), which uses the Brazilian food composition table [10]. Dietary reference intakes were used to prescribe all diets with suitable macronutrient distribution [11].

Clinical nutrition assessments performed at 15, 30, and 60 days after the start of the study confirmed the adherence to interventions, which compared the reported food intake and weight loss, as well as statin use. All participants reported full adherence to the interventions.

### 2.2. Blood Biochemical Analyses

Venous blood was drawn after a 12 h fast and put in the tubes without anticoagulant and then centrifuged for 20 min at 1800× *g* (Eppendorf, Hamburg, Germany) for serum separation.

Serum total cholesterol and triglycerides were determined by the colorimetric-enzymatic method (Cholesterol Oxidase Phenol Ampyrone-CHOD-PAP, Merck KGaA, Darmstadt, Germany). HDL-c was determined by the same method after apoB-containing lipoproteins were precipitated using precipitating reagent consisting of magnesium chloride and phosphotungstic acid. LDL-c was calculated using the Friedewald equation [12]. Serum apolipoproteins A-I (apoA-I) and B (apoB) were determined using the immunonephelometric method (ProSpec-Tany TechnoGene Ltd., Rehovot, Israel). Serum glucose was determined by the colorimetric-enzymatic method using a commercial kit (Dimension^®^ Flex Reagent Cartridge). Serum C-reactive protein (CRP) was determined by an immunoturbidimetric assay using a commercial kit (Roche Diagnostics, Manheim, Germany).

Serum Sirt-1 concentrations were determined using an ELISA kit (Uscn Life Science, Wuhan, Hubei, China). Sirt-1 samples, before and after interventions, were analyzed in duplicate and the same ELISA plate using the Multiscan FC plate reader (Thermo Fisher Scientific, Waltham, MA, USA), with a coefficient of variation of 12%, according to the manufacturer’s instructions. All analyses were performed according to the manufacturers’ instructions.

### 2.3. Lipid Transfer to HDL Assay

The lipid transfer assay was realized through an artificial lipid nanoemulsion prepared according to the technique described by Ginsburg et al. [13] and modified by Maranhão et al. [14]. In a flask, 40 mg phosphatidylcholine, 20 mg cholesterol oleate, 1 mg triolein, and 0.5 mg cholesterol were added. Subsequently, the isotopes 3H-cholesterol ester and 14C-phosphatidylcholine or 3H-triglycerides and free 14C-cholesterol were added to the lipid mixture. After the addition of 10 mL of 0.01M tris-HCl buffer, pH 8, the lipid mixture was emulsified by ultrasonic irradiation using the Branson equipment, model 450A (Arruda Ultra-Som, São Paulo, Brazil), power 125 watts, for 3 h, under nitrogen atmosphere, at a temperature ranging from 51 to 55 °C.

The lipid solution was purified in a two-step ultracentrifugation (OptimaTM XL-100K Ultracentrifuge, rotor SW-41, Beckman, Brea, CA, USA). In the first step, the material from the top of the tube, resulting from centrifugation at 200,000× *g* for 30 min at 4 °C, was removed by aspiration (1 mL) and discarded. Potassium bromide (KBr) was added to the remaining material, adjusting the density to 1.21 g/mL. After the second centrifugation (200,000× *g* for 2 h at 4 °C), the artificial lipid nanoemulsion was recovered at the top of the tube by aspiration. Excess KBr was removed by dialysis against 2 exchanges of 1000 volumes 0.01 M tris HCl buffer pH 8. Finally, the nanoemulsion was sterilized by filtration on 0.22 m (pore size) Milipore membrane under laminar flow and stored at 4 °C for up to 15 days.

The lipid transfer assay was performed as described by Lo Prete et al. [5]. A 200 µL aliquot of plasma from the participants was incubated with 50 µL of the nanoemulsion labeled with the radioactive lipids (cholesterol oleate-3H and phospholipids-14C or triolein-3H and cholesterol-14C) at 37 °C under stirring for 1 h. After this procedure, 250 µL of precipitating reagent (0.2% dextran/0.3 mol/L MgCl2) was added followed by stirring for 30 s and centrifugation for 10 min at 3000 revolutions per minute. The infranadant containing the nanoemulsion and the plasma lipoproteins containing apo-B were discarded. The supernatant, containing HDL, was subjected to the counting of radioactivity in a beta counter (Liquid Scintillation Analyzer-TRI-CARB2100TR, PerkinElmer, Massachusetts, USA) corresponding to the transfer of radioactive lipids from the nanoemulsion to the HDL of the subject.

The percentage of transfer of each of the radioactive lipids was calculated considering 100% of the total radioactivity used in the incubation.

### 2.4. Antioxidant Capacity of HDL

The antioxidant capacity of HDL was based on the lag time test previously described by Esterbauer et al. [7]. For assay preparation, a standard LDL sample was diluted in PBS without EDTA to a final concentration of 0.083 mg/mL and distributed in flat-bottom plates containing 96 wells. Subsequently, HDL samples (200 mg/mL) previously precipitated were added. Peroxidation was induced by CuSO4 (30 µmol/L), and absorbance was measured at 234 nm for 5 h. The oxidation resistance phase (lag time), the propagation phase of the conjugated dienes (indicated by the increase in absorbance), and the decomposition phase of these compounds (plateau) were observed. From the results, the lag time phase was calculated, as well as the maximum rate of lipid peroxidation (V_max_), the maximum production of conjugated dienes (DO_max_), the time for maximum production of conjugated dienes (T_max_), and the area under the curve generated (AUC).

### 2.5. Statistical Analysis

The sample size calculation below used as a reference the percentage of free cholesterol variation of free cholesterol transfer from an artificial lipid nanoemulsion to HDL observed in a previous study that included patients with premature CAD [8]. Power calculation indicated that, at n ≥ 13 in each group, we have 80% power at a significance level of 0.05 to detect a difference in lipid transfer values between groups.

Results are expressed in mean ± SD. Pre-post analysis of differences was assessed using the paired *t*-test. Differences between groups were evaluated by one-way ANOVA, using Bonferroni as a post hoc test.

To further evaluate the influence of interventions on the HDL functionality outcomes, we used multiple linear regressions using the backwards stepwise method for the identification of the strongest predictors, which resulted in the “final models” expressed in the results section. For the model construction, we used the traditional cardiometabolic risk factors (we used LDL-c and HDL-c instead of apoB and apoA-I because LDL-c and HDL-c presented higher correlations with the outcomes), serum Sirt-1, and the interventions (energy restriction and atorvastatin were used as categorical variables as “0” being without intervention and “1” being the respective intervention). To evaluate how the variables in the model were associated with each other, we built models for each variable, and thus verified their influence on the changes detected in the study. The final models were chosen using as parameters the best model’s R^2^ possible concurrently with the highest F-value. Each independent and dependent continuous variable included in the model (except the categorical variables “energy restriction” and “atorvastatin”) were used as variable changes (delta, i.e., post- minus pre-intervention).

All statistical analyses were performed using the SPSS Software version 20.0.

## 3. Results

The overall mean age of the participants was 50.5 ± 3.8 years. All baseline characteristics were similar among groups (Table 1). Overall serum Sirt-1 was 214.4 ± 101.1 pg/mL.

### 3.1. Pre-Post Analysis

Table 1 shows the post-intervention data. After dietary energy restriction, body weight, BMI, and waist circumference were reduced significantly by 1.05 kg (*p* = 0.020), 0.42 kg/m^2^ (*p* = 0.019), and 2.38 cm (*p* = 0.005), respectively. We observed a statistically significant reduction of apoB and apoA-I (*p* = 0.013) by 0.17 g/L (*p* = 0.011) and 0.13 g/L (*p* = 0.013), respectively. We also observed non-statistically significant reductions of serum total cholesterol (*p* = 0.074), HDL-c (*p* = 0.075), LDL-c (*p* = 0.072), and non-HDL-c (*p* = 0.067). The HDL particle functionality analysis showed a decrease in free and esterified cholesterol transfer to HDL particles by, respectively, 0.70% (*p* = 0.025) and 0.55% (*p* = 0.005). The HDL lipid composition and antioxidant capacity did not show statistically significant changes. The serum Sirt-1 increased by 13.5 pg/mL (*p* = 0.595) but did not reach statistical significance in this group.

In the atorvastatin group, no statistically significant differences were observed in body weight (*p* = 0.685), BMI (*p* = 0.681), and waist circumference (*p* = 0.281). As expected, we observed reductions in triglycerides, total cholesterol, non-HDL-c, LDL-c, and apoB by 67.8 mg/dL (*p* = 0.004), 61.6 mg/dL (*p* = 0.001), 65.5 mg/dL (*p* < 0.001), 54.1 mg/dL (*p* = 0.001), and 0.37 g/L (*p* = 0.001) after statin treatment, respectively. Furthermore, there was a reduction in serum Sirt-1 but it did not reach statistical significance (*p* = 0.057). HDL functionality parameters, such as lipid transfer to HDL particles, lipid composition, and antioxidant capacity, did not change.

The control group showed no changes in the parameters analyzed

### 3.2. Multiple Linear Regressions

Table 2, Table 3 and Table 4 show the multiple linear regression results of the effects of the interventions adjusted by cardiometabolic variables. Supplementary Tables S1–S3 show the initial (full) model which used all adjustment variables.

Table 2 and Table S1 show the intervention effects on cardiometabolic risk factors (BMI, serum HDL-c, LDL-c, triglycerides, and glucose) and Sirt-1. Energy restriction was associated with an increase in Sirt-1 of 63.6 pg/mL (β = 63.608; CI95% = 1.502–125.714; *p* = 0.045). BMI was positively associated with Sirt-1 (β = 36.865; CI95% = 0.804–72.926; *p* = 0.045). BMI, in turn, decreased by 0.8 kg/m^2^ after energy restriction treatment (β = −0.811; CI95% = −1.349–−0.273; *p* = 0.004). Furthermore, the Sirt–1 increase was associated with serum glucose increase (β = 2.212; CI95% = 0.309–4.114; *p* = 0.024).

Atorvastatin treatment reduced LDL-c by 40 mg/dL (β = −40.045; CI95% = −69.949–−10.140; *p* = 0.010). LDL-c reduction was also associated with triglycerides reduction (β = 0.243; CI95% = 0.043–0.443; *p* = 0.019). Triglycerides, in turn, were reduced by energy restriction (β = −61.914; CI95% = −108.758–−15.070; *p* = 0.011) and atorvastatin (β = −71.840; CI95% = −122.773–−20.907; *p* = 0.007) and presented an inverse association with HDL-c (β = −3.929; CI95% = −7.407–−0.450; *p* = 0.028).

Regarding lipid transfer to HDL particles (Table 3 and Table S2), it was found that changes (delta) in serum triglycerides (β = 0.006; CI95% = 0.002–0.009; *p* = 0.004) and HDL-c (β = 0.042; CI95% = 0.002–0.081; *p* = 0.038) were positively associated with esterified cholesterol transfer. Free cholesterol transfer was also associated with triglycerides (β = 0.007; CI95% = 0.002–0.013; *p* = 0.006) and HDL-c (β = 0.082; CI95% = 0.022–0.142; *p* = 0.009). As for HDL lipid composition, BMI reduction caused a decrease in free cholesterol (β = 0.818; CI95% = 0.044–1.593; *p* = 0.039) and the phospholipids (β = 7.486; CI95% = 0.350–14.622; *p* = 0.040) content of HDL particles. The increase in serum Sirt-1 was also associated with a reduction in the phospholipid content of HDL (β = −0.071; CI95% = −0.136–0.006; *p* = 0.033). We did not observe any associations of atorvastatin treatment or LDL-c reduction with HDL particle lipid composition or lipid transfer parameters.

We also found that serum LDL-c reduction was associated with decreased oxidation products of HDL, represented by the maximum rate of lipid peroxidation (Vmax − β = 0.002; CI95% = 0.000–0.003; *p* = 0.022), time for maximum production of conjugated dienes (Tmax – β = 0.178; CI95% = 0.027–0.330; *p* = 0.023), and maximum production of conjugated dienes (DOmax − β = 0.001; CI95% = 0.000–0.001; *p* = 0.029), but without statistically significant associations with AUC (Table 4 and Table S3). Furthermore, we did not observe statistically significant associations of HDL antioxidant capacity with serum Sirt-1, BMI, or energy restriction.

## 4. Discussion

This randomized study showed that energy restriction and atorvastatin affect the lipid profile, serum Sirt-1 concentrations, and HDL functionality differently. We observed a reduction in BMI and triglycerides and increased Sirt-1 serum concentration in the energy restriction group. We also observed in this group a decrease in free and esterified cholesterol transfer to HDL and a reduction in HDL’s phospholipid and free cholesterol content. The atorvastatin group improved HDL functionality by reducing the conjugated diene production and the transfer to HDL of free and esterified cholesterol.

Reduced Sirt-1 expression and activity were associated with worse cardiometabolic risk, such as higher blood lipids, glucose, inflammation, and adiposity [15]. Few clinical studies showed that circulating Sirt-1, which increases with energy restriction or diet bioactive compounds, is associated with higher flow-mediated vasodilation, lower plasma noradrenaline, lower blood lipids, glucose, and adiposity [16,17,18,19]. However, whether a causal relationship between risk factors and Sirt-1 exists remains unclear. Furthermore, it is debated whether circulating Sirt-1 reflects the cellular expression of Sirt-1 and what the sources of circulating Sirt-1 are. No evidence was found regarding sources of circulating Sirt-1, but previous studies showed that Sirt-1 concentration and expression are associated [20,21]. Despite that, decreased Sirt-1 serum concentrations and expression of Sirt-1 were predictive of higher size of coronary atherosclerosis plaques in asymptomatic and symptomatic patients with CAD [22,23]. Thus, the use of circulating Sirt-1 seems to be useful for clinical research, since it is easier to evaluate the concentration than the expression. Interestingly, HDL serum concentration and better function, measured by paraoxonase-1 activity, were positively associated with higher Sirt-1 expression [23]. Free cholesterol and phospholipids are found in the membrane monolayer of HDL. These lipids influence membrane fluidity, and HDL particles with reduced fluidity are prone to oxidation and, therefore, to HDL dysfunction [24,25,26,27]. A previous study showed that virgin olive oil decreased HDL-free cholesterol composition, which led to an increase in the fluidity of the HDL lipid monolayer and consequently higher HDL cholesterol efflux capacity (different than esterified cholesterol, which increases membrane fluidity) [27]. Patients with CAD showed lower phospholipids and free cholesterol transfer to HDL particles, which could lead to a lower respective composition of these lipids, which could be attributed to higher degradation of these lipids by oxidation [8]. Furthermore, phospholipid fatty acid composition also contributes to membrane fluidity, and membranes rich in polyunsaturated fatty acids, especially from the omega-3 family, contribute to reduced cardiovascular risk [28]. Therefore, our results suggest that increased Sirt-1 led to a better lipid composition of HDL (i.e., lower free cholesterol that decreases membrane fluidity), which translates into higher HDL function (i.e., higher membrane fluidity that increases reverse cholesterol transport capacity and reduces oxidation propensity). However, we did not measure membrane fluidity in our study. Our results also showed a positive association between changes in Sirt-1 and BMI. This association is poorly understood, but a previous study showed similar results in women [29].

Another study finding was the association between reduced serum triglycerides and free and esterified cholesterol transfer to HDL. Reduced free and esterified cholesterol transfer to HDL decreases reverse cholesterol transport due to decreased cholesterol ester transfer protein (CETP) concentration and activity caused by the triglycerides-lowering effect of dietary energy restriction or atorvastatin [30,31]. Despite the decrease in plasma CETP, the cholesterol efflux properties of HDL did not change after energy restriction in a previous study [30]. Our results also showed that energy restriction decreased plasma HDL-c and apoA-I. A meta-analysis of 70 studies showed that HDL reduces during weight loss, but the HDL level increases after the weight stabilization phase [32]. Our patients’ short intervention time of energy restriction may explain the low HDL plasma concentration because they did not reach the weight stabilization phase. In addition, the triglyceride reduction due to atorvastatin reached approximately 39.8%, similar to those described in the literature [31]. The reason for this marked reduction is unclear. Our study also showed that LDL-c reduction by atorvastatin decreased the generation of conjugated dienes in HDL, indicating an improvement in the antioxidant capacity of HDL. Higher LDL-c is associated with more oxidized LDL (oxLDL), an essential source of systemic oxidative stress and immunoinflammatory response characteristic of atherosclerosis. In a non-inflammatory state, HDL promotes cholesterol efflux from macrophages, inhibits the production of reactive oxygen species (ROS), and increases nitric oxide (NO) production from endothelial cells. However, in acute phase response or systemic inflammation, the latter being a hallmark of atherosclerosis, it promotes aggregation of serum amyloid A with HDL, reducing its cholesterol efflux capacity and NO production. Furthermore, the dysfunctional HDL increases ROS production, with marked production of lipid peroxides, such as conjugated dienes [33,34]. Atorvastatin also has an antioxidant effect by upregulating catalase and downregulating NAD(P)H oxidase expressions. These findings contributed to the higher antioxidant capacity of HDL [9].

Our study has some limitations. First, the small sample of patients limited the evaluation of the actual effect of the interventions. Despite that, the sample size had enough power to detect changes in HDL function. Another limitation is that we did not assess CETP to evaluate the changes in cholesterol efflux capacity better. Additionally, we did not evaluate the menstrual cycle. It is known that estrogen and progesterone levels greatly influence blood lipoprotein levels and antioxidant status [35]. And finally, the role of HDL function in predicting clinical outcomes is still unclear, and future studies are needed.

## 5. Conclusions

Our study showed that energy restriction improved HDL lipid composition and function, increased Sirt-1 serum concentration, and led to BMI reduction, which could increase membrane fluidity and reduce HDL’s propensity to oxidation. As for atorvastatin, HDL antioxidant capacity is modulated by the cholesterol-lowering effect. Further studies are needed to investigate the long-term impact of HDL function and circulating Sirt-1 on cardiovascular health.

## Figures and Tables

**Table 1:** Baseline and post-intervention characteristics of the participants.

	Energy Restriction			Atorvastatin			Control			
Variables	Baseline	Post-Intervention	*p*-Value	Baseline	Post-Intervention	*p*-Value	Baseline	Post-Intervention	*p*-Value	Baseline *p*-Value
Age, y	49 ± 3		N/A	51 ± 4		N/A	51 ± 4			0.286
Body weight (Kg)	70 ± 10	69 ± 10	0.020	69 ± 11	68 ± 11	0.685	72 ± 13	74 ± 13	0.070	0.009^a^
Body mass index, kg/m^2^	29 ± 4	28 ± 4	0.019	28 ± 3	28 ± 3	0.681	29 ± 4	30 ± 4	0.083	0.010^a^
WC, cm	92 ± 11	90 ± 10	0.050	91 ± 8	91 ± 8	0.285	96 ± 10	96 ± 10	0.886	0.012^b^
Glucose, mg/dL	100 ± 99	99 ± 10	0.693	119 ± 51	113 ± 30	0.360	111 ± 44	11 ± 40	0.871	0.475
Total cholesterol, mg/dL	229 ± 55	207 ± 59	0.074	231 ± 52	169 ± 47	0.010	224 ± 62	238 ± 48	0.341	0.001^c^
LDL-c, mg/dL	143 ± 40	129 ± 49	0.072	152 ± 43	98 ± 44	0.010	151 ± 49	155 ± 44	0.723	0.004^c^
HDL-c, mg/dL	56 ± 13	53 ± 14	0.075	45 ± 13	49 ± 13	0.087		47 ± 11	0.340	0.036
Non-HDL-c, mg/dL	173 ± 51	156 ± 57	0.200	186 ± 50	120 ± 45	<0.001	178 ± 57	187 ± 53	0.537	0.001^c^
Triglycerides, mg/dL	154 ± 100	129 ± 77	0.151	178 ± 97	111 ± 59	0.004	136 ± 60	163 ± 91	0.345	0.006^c^
ApoA-I, g/L	1.59 ± 0.32	1.43 ± 0.28	0.013	1.44 ± 0.25	1.44 ± 0.22	0.974	1.46 ± 0.22	1.49 ± 0.22	0.959	0.075
ApoB, g/L	1.20 ± 0.35	1.05 ± 0.36	0.011	1.20 ± 0.30	0.87 ± 0.28	0.010	1.20 ± 0.36	1.21 ± 0.31	0.640	0.003^c^
Lipoprotein (a), mg/dL	35.70 ± 31.4	40.51 ± 31.96	0.204	50.52 ± 33	56.93 ± 38.81	0.100	72 ± 39	67 ± 39	0.895	0.382
Lipid transfer to HDL, %										
Esterified cholesterol	4.70 ± 0.75	4.15 ± 0.55	0.005	4.37 ± 0.70	4.13 ± 0.38	0.257	4.63 ± 0.52	4.61 ± 0.50	0.925	0.151
Free cholesterol	5.60 ± 1.32	4.90 ± 1.03	0.025	5.16 ± 0.89	4.97 ± 0.53	0.460	5.33 ± 0.76	5.39 ± 0.68	0.857	0.173
Lipid composition of HDL, %										
Esterified cholesterol	40.24 ± 9.89	42.13 ± 16	0.675	41.32 ± 10.51	42.47 ± 17.36	0.858	33.23 ± 16.17	27.08 ± 16.50	0.357	0.559
Free cholesterol	4.52 ± 1.16	5.02 ± 1.82	0.355	4.53 ± 1.04	4.49 ± 1.23	0.918	5.09 ± 1.24	5.00 ± 1.14	0.859	0.630
Phospholipids	47.96 ± 8.89	44.09 ± 12.91	0.314	47.72 ± 9.21	46.26 ± 14.25	0.788	52.85 ± 13.24	57.62 ± 16.14	0.421	0.457
Triglycerides	7.21 ± 2.08	8.76 ± 5.33	0.331	6.42 ± 1.69	6.78 ± 3.02	0.662	8.83 ± 4.76	10.29 ± 6.05	0.345	0.775
Sirt-1, pg/mL	206 ± 76	220 ± 82	0.595	224 ± 89	165 ± 61	0.057	213 ± 136	201 ± 91	0.538	0.122

Pre-post tests were done using paired *t*-test. Comparisons between groups were done by using one-way ANOVA adjusted by Bonferroni method.

**Table 2:** Effects of interventions and variables’ changes on risk factors and serum sirtuin-1.

	Final Model				
Variables	R^2^	β	95% CI for β		*p*-Value
			Lower	Upper	
*Serum sirtuin-1*	0.308				
Constant		−32.460	−68.365	3.446	0.075
BMI, kg/m^2^		36.865	0.804	72.926	**0.045**
Serum HDL-c, mg/dL					
Serum LDL-c, mg/dL					
Serum triglycerides, mg/dL					
Serum glucose, mg/dL		2.212	0.309	4.114	**0.024**
Energy restriction		63.608	1.502	125.714	**0.045**
Atorvastatin					
*Serum LDL-c*	0.396				
Constant		−10.909	−26.252	4.433	0.157
Serum sirtuin-1, pg/mL					
BMI, kg/m^2^					
Serum HDL-c, mg/dL					
Serum triglycerides, mg/dL		0.243	0.043	0.443	**0.019**
Serum glucose, mg/dL					
Energy restriction					
Atorvastatin		−40.045	−69.949	−10.140	**0.010**
*Serum HDL-c*	0.178				
Constant		−1.576	−3.483	0.331	0.102
Serum sirtuin-1, pg/mL					
BMI, kg/m^2^					
Serum LDL-c, mg/dL					
Serum triglycerides, mg/dL		−0.036	−0.064	−0.009	**0.010**
Serum glucose, mg/dL					
Energy restriction					
Atorvastatin					
*Serum triglycerides*	0.382				
Constant		27.166	−7.574	61.905	0.121
Serum sirtuin-1, pg/mL					
BMI, kg/m^2^					
Serum HDL-c, mg/dL		−3.929	−7.407	−0.450	**0.028**
Serum LDL-c, mg/dL					
Serum glucose, mg/dL					
Energy restriction		−61.914	−108.758	−15.070	**0.011**
Atorvastatin		−71.840	−122.773	−20.907	**0.007**
*Serum glucose*	0.174				
Constant		−1.239	−5.910	3.432	0.593
Serum sirtuin-1, pg/mL		0.067	0.016	0.117	**0.011**
BMI, kg/m^2^					
Serum HDL-c, mg/dL					
Serum LDL-c, mg/dL					
Serum triglycerides, mg/dL					
Energy restriction					
Atorvastatin					
*BMI*	0.270				
Constant		0.340	0.014	0.666	**0.042**
Serum sirtuin-1, pg/mL		0.003	0.000	0.006	**0.026**
Serum HDL-c, mg/dL					
Serum LDL-c, mg/dL					
Serum triglycerides, mg/dL					
Serum glucose, mg/dL					
Energy restriction		−0.811	−1.349	−0.273	**0.004**
Atorvastatin					

**Table 3:** HDL particle’s lipid composition and cholesterol transfer.

	Final Model				
Variables	R^2^	β	95% CI for β		*p*-Value
			Lower	Upper	
*Esterified cholesterol transfer*	0.358				
Constant		−0.063	−0.439	0.312	0.733
Serum sirtuin-1, pg/mL					
BMI, kg/m^2^					
Serum HDL-c, mg/dL		0.042	0.002	0.081	**0.038**
Serum LDL-c, mg/dL					
Serum triglycerides, mg/dL		0.006	0.002	0.009	**0.004**
Serum glucose, mg/dL					
Energy restriction		−0.243	−0.782	0.297	0.366
Atorvastatin		−0.074	−0.668	0.520	0.801
*Free cholesterol transfer*	0.257				
Constant		−0.105	−0.447	0.237	0.538
Serum sirtuin-1, pg/mL					
BMI, kg/m^2^					
Serum HDL-c, mg/dL		0.082	0.022	0.142	**0.009**
Serum LDL-c, mg/dL					
Serum triglycerides, mg/dL		0.007	0.002	0.013	**0.006**
Serum glucose, mg/dL					
Energy restriction					
Atorvastatin					
*HDL particle’s free cholesterol*	0.189				
Constant		−0.675	−1.764	0.413	0.215
Serum sirtuin-1, pg/mL					
BMI, kg/m^2^		0.818	0.044	1.593	**0.039**
Serum HDL-c, mg/dL		−0.068	−0.170	0.033	0.179
Serum LDL-c, mg/dL					
Serum triglycerides, mg/dL					
Serum glucose, mg/dL					
Energy restriction		1.363	−0.181	2.907	0.082
Atorvastatin		0.907	−0.642	2.456	0.241
*HDL particle’s esterified cholesterol*	0.119				
Constant		−3.591	−16.910	9.729	0.586
Serum sirtuin-1, pg/mL					
BMI, kg/m^2^		−6.501	−16.289	3.286	0.185
Serum HDL-c, mg/dL					
Serum LDL-c, mg/dL					
Serum triglycerides, mg/dL					
Serum glucose, mg/dL		0.314	−0.190	0.819	0.213
Energy restriction		2.967	−16.446	22.381	0.757
Atorvastatin		4.159	−14.770	23.089	0.657
*HDL particle’s phospholipids*	0.187				
Constant		−0.410	−6.230	5.410	0.887
Serum sirtuin-1, pg/mL		−0.071	−0.136	−0.006	**0.033**
BMI, kg/m^2^		7.486	0.350	14.622	**0.040**
Serum HDL-c, mg/dL					
Serum LDL-c, mg/dL					
Serum triglycerides, mg/dL					
Serum glucose, mg/dL					
Energy restriction					
Atorvastatin					
*HDL particle’s triglycerides*	0.087				
Constant		1.206	−2.376	4.788	0.496
Serum sirtuin-1, pg/mL		0.008	−0.016	0.032	0.511
BMI, kg/m^2^		0.379	−2.216	2.974	0.767
Serum HDL-c, mg/dL		0.067	−0.288	0.423	0.700
Serum LDL-c, mg/dL		0.003	−0.048	0.053	0.912
Serum triglycerides, mg/dL		0.015	−0.020	0.050	0.387
Serum glucose, mg/dL		−0.044	−0.184	0.096	0.527
Energy restriction		0.970	−4.594	6.534	0.723
Atorvastatin		−0.060	−6.131	6.012	0.984

**Table 4:** HDL particle’s antioxidant capacity.

	Final Model				
Variables	R^2^	β	95% CI for β		*p*-Value
			Lower	Upper	
*VMax*	0.238				
Constant		0.068	−0.016	0.151	0.110
Serum sirtuin-1, pg/mL					
BMI, kg/m^2^					
Serum HDL-c, mg/dL		0.011	0.000	0.022	0.052
Serum LDL-c, mg/dL		0.002	0.000	0.003	**0.022**
Serum triglycerides, mg/dL					
Serum glucose, mg/dL					
Energy restriction					
Atorvastatin					
*TMax*	0.212				
Constant		3.892	−6.564	14.348	0.450
Serum sirtuin-1, pg/mL					
BMI, kg/m^2^		4.941	−4.398	14.280	0.286
Serum HDL-c, mg/dL					
Serum LDL-c, mg/dL		0.178	0.027	0.330	**0.023**
Serum triglycerides, mg/dL		−0.057	−0.144	0.030	0.188
Serum glucose, mg/dL					
Energy restriction		3.181	−11.279	17.641	0.654
Atorvastatin		5.155	−10.590	20.901	0.506
*Optic density peak*	0.230				
Constant		0.014	−0.023	0.051	0.432
Serum sirtuin-1, pg/mL					
BMI, kg/m^2^					
Serum HDL-c, mg/dL		0.002	−0.001	0.006	0.178
Serum LDL-c, mg/dL		0.001	0.000	0.001	**0.029**
Serum triglycerides, mg/dL					
Serum glucose, mg/dL					
Energy restriction		0.005	−0.042	0.053	0.822
Atorvastatin		0.013	−0.044	0.070	0.637
*Area under the curve*	0.103				
Constant		158.209	−187.402	503.820	0.356
Serum sirtuin-1, pg/mL					
BMI, kg/m^2^					
Serum HDL-c, mg/dL					
Serum LDL-c, mg/dL		5.429	−0.778	11.636	0.084
Serum triglycerides, mg/dL					
Serum glucose, mg/dL					
Energy restriction					
Atorvastatin					

## Data Availability

Data is contained within the article and Supplementary Material.

## Supplementary Materials

The following supporting information can be downloaded at: https://www.mdpi.com/article/10.3390/antiox11122363/s1, Table S1. Effects of interventions and variables’ changes on risk factors and serum sirtuin-1 (full model), Table S2. HDL particle’s lipid composition and cholesterol transfer (full model), Table S3. HDL particle’s antioxidant capacity (full model).
