# Supplementary material for: The Interplay of Sirtuin-1, LDL-Cholesterol, and HDL Function: A Randomized Controlled Trial Comparing the Effects of Energy Restriction and Atorvastatin on Women with Premature Coronary Artery Disease
Source: Antioxidants (Basel). 2022 Nov 29;11(12):2363. doi: 10.3390/antiox11122363 (PMC9774144; doi:10.3390/antiox11122363)
Supplement: Supplementary file 1 [file antioxidants-11-02363-s001.zip › antioxidants-2002311-supplementary.pdf]

Supplementary tables

Table S1. Effects of interventions and variables' changes on risk factors and serum sirtuin-1 (full model).

Table S2. HDL particle's lipid composition and cholesterol transfer (full model).

Table S3. HDL particle's antioxidant capacity (full model).

Table S1. Effects of interventions and variables' changes on risk factors and serum sirtuin-1 (full model).

		Full Model			
**Variables**	**R^2**	**β**	**95% CI for β**		**p-value**
			**Lower**	**Upper**	
Serum sirtuin-1	0.341				
Constant		-29.798	-86.330	26.735	0.289
BMI, kg/m²		34.537	-5.073	74.147	0.085
Serum HDL-c, mg/dL		-1.084	-6.795	4.628	0.701
Serum LDL-c, mg/dL		0.133	-0.682	0.948	0.741
Serum triglycerides, mg/dL		0.139	-0.419	0.697	0.613
Serum glucose, mg/dL		2.166	0.070	4.262	**0.043**
Energy restriction		63.882	-22.267	150.031	0.140
Atorvastatin		3.920	-93.872	101.712	0.935
Serum LDL-c	0.428				
Constant		1.999	-25.338	29.335	0.882
Serum sirtuin-1, pg/mL		0.030	-0.153	0.213	0.741
BMI, kg/m²		-7.612	-27.207	11.982	0.433
Serum HDL-c, mg/dL		-0.035	-2.750	2.679	0.979
Serum triglycerides, mg/dL		0.193	-0.062	0.448	0.133
Serum glucose, mg/dL		0.034	-1.036	1.104	0.949
Energy restriction		-24.570	-65.973	16.834	0.234
Atorvastatin		-53.835	-95.244	-12.427	**0.013**
Serum HDL-c	0.290				
Constant		-1.232	-5.102	2.639	0.520
Serum sirtuin-1, pg/mL		-0.005	-0.031	0.021	0.701
BMI, kg/m²		0.286	-2.538	3.109	0.837
Serum LDL-c, mg/dL		-0.001	-0.056	0.054	0.979
Serum triglycerides, mg/dL		-0.036	-0.071	-0.001	**0.045**
Serum glucose, mg/dL		0.104	-0.043	0.251	0.158
Energy restriction		-1.873	-7.888	4.142	0.529
Atorvastatin		1.557	-5.027	8.140	0.632
Serum triglycerides	0.450				
Constant		24.572	-14.100	63.245	0.204
Serum sirtuin-1, pg/mL		0.066	-0.200	0.332	0.613
BMI, kg/m²		1.829	-27.019	30.676	0.898
Serum HDL-c, mg/dL		-3.757	-7.433	-0.082	**0.045**
Serum LDL-c, mg/dL		0.409	-0.132	0.950	0.133
Serum glucose, mg/dL		0.315	-1.239	1.869	0.681
Energy restriction		-49.066	-107.944	9.812	0.099
Atorvastatin		-37.427	-103.363	28.509	0.255
Serum glucose	0.242				
Constant		1.778	-8.090	11.646	0.715
Serum sirtuin-1, pg/mL		0.064	0.002	0.125	**0.043**
BMI, kg/m²		-0.497	-7.662	6.668	0.888
Serum HDL-c, mg/dL		0.671	-0.276	1.618	0.158
Serum LDL-c, mg/dL		0.004	-0.136	0.144	0.949
Serum triglycerides, mg/dL		0.019	-0.076	0.115	0.681
Energy restriction		-1.455	-16.811	13.901	0.847
Atorvastatin		-4.664	-21.334	12.007	0.571
BMI	0.321				
Constant		0.530	0.037	1.023	**0.036**
Serum sirtuin-1, pg/mL		0.003	0.000	0.006	0.085
Serum HDL-c, mg/dL		0.005	-0.048	0.058	0.837
Serum LDL-c, mg/dL		-0.003	-0.010	0.005	0.433
Serum triglycerides, mg/dL		0.000	-0.005	0.006	0.898
Serum glucose, mg/dL		-0.001	-0.022	0.019	0.888
Energy restriction		-1.046	-1.770	-0.321	**0.006**
Atorvastatin		-0.586	-1.463	0.291	0.182

Table S2. HDL particle's lipid composition and cholesterol transfer (full model).

	Full Model				
**Variables**	**R^2**	**β**	**95% CI for β**		**p-value**
			**Lower**	**Upper**	
Esterified cholesterol transfer	0.381				
Constant		-0.084	-0.517	0.349	0.694
Serum sirtuin-1, pg/mL		0.001	-0.002	0.004	0.581
BMI, kg/m²		0.067	-0.246	0.381	0.663
Serum HDL-c, mg/dL		0.042	-0.001	0.085	0.056
Serum LDL-c, mg/dL		0.002	-0.005	0.008	0.599
Serum triglycerides, mg/dL		0.005	0.001	0.009	**0.017**
Serum glucose, mg/dL		-0.002	-0.019	0.015	0.844
Energy restriction		-0.180	-0.852	0.493	0.588
Atorvastatin		0.061	-0.673	0.794	0.867
Free cholesterol transfer	0.315				
Constant		0.055	-0.642	0.751	0.873
Serum sirtuin-1, pg/mL		0.001	-0.003	0.006	0.558
BMI, kg/m²		0.122	-0.383	0.627	0.623
Serum HDL-c, mg/dL		0.071	0.002	0.140	**0.045**
Serum LDL-c, mg/dL		0.000	-0.010	0.010	0.967
Serum triglycerides, mg/dL		0.006	-0.001	0.012	0.095
Serum glucose, mg/dL		0.003	-0.024	0.030	0.818
Energy restriction		-0.402	-1.484	0.681	0.453
Atorvastatin		-0.090	-1.271	1.091	0.877
HDL particle's free cholesterol	0.236				
Constant		-0.607	-1.805	0.591	0.308
Serum sirtuin-1, pg/mL		-0.001	-0.009	0.007	0.827
BMI, kg/m²		0.921	0.053	1.789	**0.038**
Serum HDL-c, mg/dL		-0.082	-0.201	0.037	0.169
Serum LDL-c, mg/dL		0.008	-0.009	0.025	0.317
Serum triglycerides, mg/dL		-0.005	-0.017	0.006	0.348
Serum glucose, mg/dL		-0.003	-0.050	0.044	0.901
Energy restriction		1.378	-0.483	3.239	0.140
Atorvastatin		1.051	-0.979	3.082	0.298
HDL particle's esterified cholesterol	0.217				
Constant		0.569	-14.059	15.198	0.937
Serum sirtuin-1, pg/mL		0.071	-0.027	0.170	0.147
BMI, kg/m²		-9.366	-19.966	1.233	0.081
Serum HDL-c, mg/dL		0.266	-1.186	1.718	0.710
Serum LDL-c, mg/dL		-0.075	-0.282	0.132	0.464
Serum triglycerides, mg/dL		-0.039	-0.181	0.103	0.579
Serum glucose, mg/dL		0.157	-0.415	0.729	0.578
Energy restriction		-5.612	-28.336	17.112	0.616
Atorvastatin		-4.196	-28.993	20.601	0.731
HDL particle's phospholipids	0.254				
Constant		-1.165	-13.526	11.195	0.848
Serum sirtuin-1, pg/mL		-0.078	-0.161	0.005	0.065

BMI, kg/m²		8.057	-0.899	17.013	0.076
Serum HDL-c, mg/dL		-0.254	-1.481	0.973	0.674
Serum LDL-c, mg/dL		0.064	-0.111	0.239	0.462
Serum triglycerides, mg/dL		0.029	-0.091	0.149	0.621
Serum glucose, mg/dL		-0.111	-0.595	0.373	0.642
Energy restriction		3.311	-15.890	22.513	0.726
Atorvastatin		3.228	-17.725	24.180	0.754
HDL particle's triglycerides	0.087				
Constant		1.206	-2.376	4.788	0.496
Serum sirtuin-1, pg/mL		0.008	-0.016	0.032	0.511
BMI, kg/m²		0.379	-2.216	2.974	0.767
Serum HDL-c, mg/dL		0.067	-0.288	0.423	0.700
Serum LDL-c, mg/dL		0.003	-0.048	0.053	0.912
Serum triglycerides, mg/dL		0.015	-0.020	0.050	0.387
Serum glucose, mg/dL		-0.044	-0.184	0.096	0.527
Energy restriction		0.970	-4.594	6.534	0.723
Atorvastatin		-0.060	-6.131	6.012	0.984

Table S3. HDL particle's antioxidant capacity (full model).

		Full Model			
Variables	R^2	β	95% CI for β		p-value
			Lower	Upper	
VMax	0.304				
Constant		0.075	-0.070	0.221	0.294
Serum sirtuin-1, pg/mL		0.000	-0.001	0.001	0.615
BMI, kg/m²		0.052	-0.102	0.205	0.492
Serum HDL-c, mg/dL		0.011	-0.005	0.026	0.175
Serum LDL-c, mg/dL		0.002	0.000	0.004	0.060
Serum triglycerides, mg/dL		0.000	-0.002	0.001	0.596
Serum glucose, mg/dL		-0.001	-0.007	0.005	0.689
Energy restriction		-0.006	-0.218	0.207	0.955
Atorvastatin		0.013	-0.205	0.232	0.899
TMax	0.227				
Constant		4.135	-7.206	15.476	0.457
Serum sirtuin-1, pg/mL		-0.002	-0.080	0.075	0.955
BMI, kg/m²		5.420	-6.543	17.383	0.357
Serum HDL-c, mg/dL		0.358	-0.874	1.590	0.553
Serum LDL-c, mg/dL		0.170	-0.002	0.341	0.053
Serum triglycerides, mg/dL		-0.041	-0.151	0.068	0.444
Serum glucose, mg/dL		-0.010	-0.476	0.456	0.964
Energy restriction		4.289	-12.303	20.880	0.597
Atorvastatin		4.374	-12.692	21.440	0.600
Optic density peak	0.244				
Constant		0.017	-0.026	0.059	0.425
Serum sirtuin-1, pg/mL		0.000	0.000	0.000	0.950
BMI, kg/m²		0.008	-0.037	0.053	0.714
Serum HDL-c, mg/dL		0.002	-0.003	0.007	0.401
Serum LDL-c, mg/dL		0.001	0.000	0.001	0.054
Serum triglycerides, mg/dL		0.000	0.000	0.000	0.714
Serum glucose, mg/dL		0.000	-0.001	0.002	0.665

Energy restriction		0.004	-0.058	0.067	0.885
Atorvastatin		0.014	-0.050	0.078	0.648
Area under the curve	0.174				
Constant		215.151	-421.240	851.542	0.490
Serum sirtuin-1, pg/mL		0.205	-4.145	4.555	0.923
BMI, kg/m^2		18.952	-652.315	690.220	0.954
Serum HDL-c, mg/dL		20.817	-48.320	89.955	0.538
Serum LDL-c, mg/dL		6.981	-2.657	16.619	0.147
Serum triglycerides, mg/dL		-0.285	-6.430	5.860	0.924
Serum glucose, mg/dL		5.801	-20.341	31.942	0.649
Energy restriction		-52.767	-983.762	878.228	0.907
Atorvastatin		169.693	-787.922	1127.309	0.716
